# Molecular Analysis of S-morphology Aflatoxin Producers From the United States Reveals Previously Unknown Diversity and Two New Taxa

**DOI:** 10.3389/fmicb.2020.01236

**Published:** 2020-06-11

**Authors:** Pummi Singh, Kenneth A. Callicott, Marc J. Orbach, Peter J. Cotty

**Affiliations:** ^1^School of Plant Sciences, The University of Arizona, Tucson, AZ, United States; ^2^United States Department of Agriculture, Agricultural Research Service, Tucson, AZ, United States; ^3^School of Food Science and Engineering, Ocean University of China, Qingdao, China

**Keywords:** aflatoxin, *Aspergillus* section *Flavi*, S-morphology, molecular phylogenetics, *Aspergillus agricola*, *Aspergillus toxicus*

## Abstract

Aflatoxins are highly toxic carcinogens that detrimentally influence profitability of agriculture and the health of humans and domestic animals. Several phylogenetically distinct fungi within *Aspergillus* section *Flavi* have S-morphology (average sclerotial size < 400 μm), and consistently produce high concentrations of aflatoxins in crops. S-morphology fungi have been implicated as important etiologic agents of aflatoxin contamination in the United States (US), but little is known about the diversity of these fungi. The current study characterized S-morphology fungi (*n* = 494) collected between 2002 and 2017, from soil and maize samples, in US regions where aflatoxin contamination is a perennial problem. Phylogenetic analyses based on sequences of the calmodulin (1.9 kb) and nitrate reductase (2.1 kb) genes resolved S-morphology isolates from the US into four distinct clades: (1) *Aspergillus flavus* S-morphotype (89.7%); (2) *Aspergillus agricola* sp. nov. (2.4%); (3) *Aspergillus texensis* (2.2%); and (4) *Aspergillus toxicus* sp. nov. (5.7%). All four S-morphology species produced high concentrations of aflatoxins in maize at 25, 30, and 35°C, but only the *A. flavus* S-morphotype produced unacceptable aflatoxin concentrations at 40°C. Genetic typing of *A. flavus* S isolates using 17 simple sequence repeat markers revealed high genetic diversity, with 202 haplotypes from 443 isolates. Knowledge of the occurrence of distinct species and haplotypes of S-morphology fungi that are highly aflatoxigenic under a range of environmental conditions may provide insights into the etiology, epidemiology, and management of aflatoxin contamination in North America.

## Introduction

The ubiquitous filamentous fungus *Aspergillus flavus* link (Ascomycota, Eurotiales) thrives on organic matter associated with both crops and non-cultivated plants (Ashworth et al., 1969; Stephenson and Russell, 1974; Kachapulula et al., 2019). *A. flavus* and its closely related species within section *Flavi* pose a serious economic threat to agriculture due to the production of the highly toxic and carcinogenic aflatoxins, which are deleterious to human and animal health (Park and Liang, 1993; Henry et al., 1999).

Aflatoxin producers within *Aspergillus* section *Flavi* can infect a wide range of food and feed crops, such as maize, sorghum, groundnuts, cottonseed, chilies, tree nuts, and insects (Shotwell et al., 1969; Shetty and Bhat, 1997; Rodrigues et al., 2009; Kachapulula et al., 2017, 2018; Singh and Cotty, 2017; Ortega-Beltran et al., 2018). Infection frequently results in contamination of the host with aflatoxins, both through ramification of hyphae in host tissue, and production of immense numbers of aflatoxin-containing conidia (Mehl and Cotty, 2010). Chronic exposure to sub-lethal concentrations of aflatoxins, through consumption of contaminated food or feed, may result in immune suppression (Jiang et al., 2005; Owaga et al., 2011), stunting (Cardeilhac et al., 1970; Gong et al., 2004), and/or hepatocellular carcinomas (Henry et al., 1999; Liu and Wu, 2010). Acute poisoning can cause severe liver damage followed by rapid death (Krishnamachari et al., 1975; CDC, 2004; Kamala et al., 2018). Strict monitoring and enforcement of regulations on the maximum allowable aflatoxin concentrations in food and feed limit exposure in developed nations (Robens and Cardwell, 2003). Monetary losses due to aflatoxins in the United States (US) are largely associated with market loss in the form of reduced prices for crops and disposal of large quantities of contaminated food (Cotty et al., 2008; Wu and Guclu, 2012). The economic repercussions can be significant, with annual losses in the US estimated to exceed $500 million annually (Robens and Cardwell, 2003). Losses for maize alone range from $52 million to $2 billion (Mitchell et al., 2016).

*Aspergillus flavus* and *Aspergillus parasiticus* are frequently implicated as primary causal agents of aflatoxin contamination of crops (Klich, 2007). *A. flavus* was delineated into the L and S morphotypes three decades ago (Cotty, 1989). When grown on the same substrate, the two morphotypes differ in sporulation; the L-morphotype produces fewer large (>400 μm) sclerotia and abundant conidia, while the S-morphotype produces copious smaller sclerotia (<400 μm) and fewer conidia (Cotty, 1989). The ability of L-morphotype isolates to produce aflatoxins varies, with L-morphotype isolates producing no aflatoxins (non-aflatoxigenic), or low to very high levels of aflatoxins (Bayman and Cotty, 1993; Mehl et al., 2012), while S-morphotype isolates consistently produce high concentrations of aflatoxins (Cotty, 1989; Probst et al., 2012; Singh and Cotty, 2019). *Aspergillus* section *Flavi* contains several described species that are morphologically similar to, but phylogenetically distinct from, the S-morphotype of *A. flavus.* While the significance of many of the newly described S-morphology species to crop contamination is unclear, the S-morphotype of *A. flavus* has been linked to severe contamination of maize and cottonseed in the US (Cotty, 1996; Jaime-Garcia and Cotty, 2004; Jaime-Garcia and Cotty, 2006; Cotty et al., 2008), and *Aspergillus aflatoxiformans* (previously reported as unnamed taxon S_BG_ or *Aspergillus parvisclerotigenus*), *Aspergillus minisclerotigenes*, and a separate unnamed lineage from Kenya [referred to as the Lethal Aflatoxicosis Fungus (LAF)] have been associated with crop contamination in Sub-Saharan Africa (Cardwell and Cotty, 2002; Frisvad et al., 2005; Probst et al., 2012, 2014; Singh and Cotty, 2019). Crop infection by S-morphology fungi is of concern because these fungi are notorious for production of high concentrations of aflatoxins and can cause unacceptable contamination even at low incidences (Cardwell and Cotty, 2002; Cotty et al., 2008; Probst et al., 2010; Singh and Cotty, 2019). The unnamed lineage LAF was responsible for the deadly aflatoxicosis outbreaks in Kenya from 2004 to 2006 that claimed the lives of more than 100 people (Probst et al., 2007, 2012). Despite the toxic potential of S-morphology fungi, many regions with significant aflatoxin contamination lack studies on endemic occurrence and diversity of these fungi. Probst et al. (2014) reported the region-specific occurrence of distinct S-morphology fungi across Sub-Saharan Africa, with *A. aflatoxiformans* (reported as Strain S_BG_) only detected in West Africa, and LAF (reported as new lineage S_B_) and *A. minisclerotigenes* confined to Eastern and Southern Africa. However, *A. minisclerotigenes* has recently been reported from dried red chilies in West Africa (Singh and Cotty, 2019).

In the US, aflatoxin contamination of several susceptible crops, including maize, almond, pistachios, fig, and groundnut is of significant economic concern, with some warm regions of the US having contamination most years (Jaime-Garcia and Cotty, 2003; Mitchell et al., 2016). Environmental conditions (high temperatures and sub-arid to arid conditions) in these regions favor growth and proliferation of aflatoxigenic fungi and crop contamination (Cotty and Jaime-Garcia, 2007). Even though fungi with S-morphology are associated with crop contamination in the US (Cotty et al., 2008), characterization of the genetic diversity of these highly aflatoxigenic fungi is unexplored.

The current study sought to (i) use molecular tools to characterize fungi with S-morphology within *Aspergillus* section *Flavi* recovered from soil and maize samples, collected from across a wide section of the southern US, (ii) assess phylogenetic relationships of US S-morphology isolates to previously described species of aflatoxin-producing fungi, (iii) determine the potential significance of the observed S-morphology fungi to aflatoxin contamination events, and (iv) test the utility of simple sequence repeat (SSR) markers for assessment of diversity among closely related S-morphology genotypes. Two novel aflatoxin producing species were discovered in the current study and are described. Resulting insights on the occurrence of distinct communities of highly aflatoxigenic S-morphology fungi may facilitate efforts to improve aflatoxin management in the US.

## Materials and Methods

### Fungal Isolates

**FIGURE 1:**
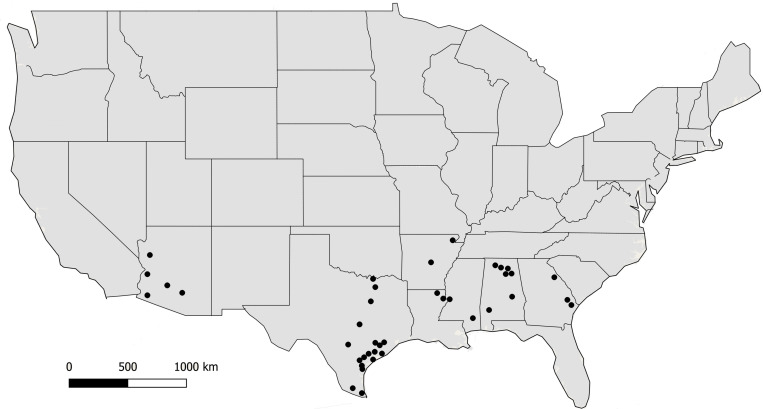
Locations where soil and maize samples were collected from 2002 to 2017. Filled circles indicate counties in each state.

Fungal isolates (*n* = 494) used in this study were recovered from soil and maize in Arizona, Texas, and the southeastern US (Figure 1). Isolates are available in the culture collection at the USDA-ARS Aflatoxin Reduction Laboratory in Tucson, Arizona. Isolates originated from soils collected in Arizona from 1989 to 2005, maize and soil samples collected in Texas from 2004 to 2014, and maize collected in the southeastern US from 2015 to 2017 (Table 1). Several reference isolates with known affiliations to already described species/lineages within *Aspergillus* section *Flavi* were obtained from the ARS Culture Collection, Peoria, IL, United States, the American Type Culture Collection, Manassas, VA, United States, or the USDA-ARS Aflatoxin Reduction Laboratory culture collection in Tucson, AZ, United States (Supplementary Table S1).

**TABLE 1:** Isolate information for 494 S-morphology isolates from *Aspergillus* section *Flavi* used in the current study.

**State**	**Source**	**Total Isolates**
Alabama	Maize	27
Arizona	Soil (Cotton)	145
Arkansas	Maize	7
Georgia	Maize	5
Louisiana	Maize	25
Mississippi	Maize	4
Texas	Maize	30
Texas	Soil (Maize)	251

### DNA Extraction and Gene Amplification

Isolates were grown on V8-Salt agar (5% V-8 juice; 2% NaCl, 2% agar; pH 6.0) for 7 d at 31°C in the dark, and DNA was extracted from the conidia as described previously (Callicott and Cotty, 2015). The concentration of stock DNA was quantified and diluted to 5 ng μl^–1^ for PCR. Deletions in the *norB-cypA* region of the aflatoxin biosynthesis gene cluster, resulting in loss of G aflatoxin production, were determined using primer sets AP1729-3551 (Ehrlich et al., 2004) and CP5F-R (Probst et al., 2012) as described previously. Amplicons were visualized on 1% agarose gels with 1 kb Plus ladder (Thermo Scientific, Waltham, MA, United States) for sizing. Positive controls included *A. parasiticus* (no deletion), *A. flavus* L and S strain morphotypes (0.9 or 1.5 kb deletions) and the previously reported LAF (2.2 kb deletion).

Partial fragments of calmodulin [*cmdA* on chromosome 2 of the *Aspergillus oryzae* RIB40 genome (Yasui et al., 1995)] and nitrate reductase (*niaD* on chromosome 4) genes were amplified with three sets of primers covering approximately 1.9 and 2.1 kb of *cmdA* and *niaD*, respectively (Table 2). The primer pair cmd3F-3R was designed based on the genome sequence of *A. flavus* NRRL 3357 (GenBank accession no. AAIH02000003) using Primer3 version 0.4.0 (Koressaar and Remm, 2007; Untergasser et al., 2012). PCR was performed in 20 μl volumes using 5 ng of genomic DNA with a PCR premix (AccuPower^®^ HotStart, Bioneer, Alameda, CA, United States) on a MyCycler thermocycler (Bio-Rad Laboratories, Richmond, CA, United States) under the following conditions: 5 min at 94°C followed by 38 cycles of 94°C for 20 s, locus-specific annealing temperatures for 30 s (Table 2), and 72°C for 1 min, and a final extension for 5 min at 72°C. Amplicons were subjected to bidirectional sequencing at the University of Arizona Genetics Core Facility (UAGC, Tucson, AZ, United States) using the amplification primers (Table 2).

**TABLE 2:** Primers and locus-specific annealing temperatures (T_*a*_) for PCR amplifications of target gene fragments from *Aspergillus* section *Flavi*.

**Primer Pair**	**Target Gene**	**Sequence**	**T_a_ (°C)**	**Reference**
cmd42-637		F-GGCCTTCTCCCTATTCGTAA	56	Probst et al., 2012
		R-CTCGCGGATCATCTCATC		
cmd2F-2R	*cmdA*	F-GGCTGGATGTGTGTAAATC	48	Probst et al., 2012
		R-ATTGGTCGCATTTGAAGGG		
cmd3F-3R		F-GTTAGTGGTTAGTCGCAG	50	Current Study
		R-CTTCAGCTCTCTGGAATC		
niaDF-AR		F-CGGACGATAAGCAACAACAC	52	Probst et al., 2012
		R-GGATGAACACCCGTTAATCTGA		
niaDBF-BR	*niaD*	F-ACGGCCGACAGAAGTGCTGA	57	Probst et al., 2012
		R-TGGGCGAAGAGACTCCCCGT		
niaDCF-CR		F-GCAGCCCAATGGTCACTACGGC	55	Singh and Cotty, 2019
		R-GGCTGCACGCCCAATGCTTC		
AP1729-3551	*norB-cypA*	F-GTGCCCAGCATCTTGGT CCACC	58	Ehrlich et al., 2004
		R-AAGGACTTGATGATTCCTC		
CP5F-R	*norB-cypA*	F-GGGACCCTTTTCCGGTGCGG	62	Probst et al., 2012
		R-GGCGGCCCCTCAGCAAACAT		

### DNA Sequence Data and Phylogenetics

Fungi with S-morphology collected from across the US, and isolates of previously described species, were used for phylogenetic comparisons. Bidirectional sequences of *cmdA* (1.9 kb) and *niaD* (2.1 kb) were used to create a consensus sequence for each amplicon by assembling six reads per gene with visual inspection and alignment using the MUSCLE algorithm within Geneious Pro Version 7.1.9 (Biomatters Ltd., Auckland, New Zealand). DNA sequence alignments were refined manually. Individual and concatenated phylogenies were constructed for both loci using Bayesian inference with 10 million generations (MrBayes version 3.2.6; Huelsenbeck and Ronquist, 2001) and maximum likelihood (ML) analyses with PhyML at Phylogeny.fr (Dereeper et al., 2008, 2010) to confirm tree topologies. Data sets were bootstrapped with 500 replicates for ML analysis. Trees were mid-point rooted using FigTree v.1.4.3 (Rambaut, 2012). Sequences from the current study have been deposited in GenBank (Supplementary Table S2).

### SSR Genotyping and Genetic Diversity

*Aspergillus flavus* S-morphotype isolates (*n* = 443) were subjected to DNA fingerprinting using 17 SSR loci markers from eight chromosomes of *A. flavus* according to Grubisha and Cotty (2009) and Islam et al. (2018). Simple sequence repeat multiplex PCR and genotyping were conducted as previously described (Grubisha and Cotty, 2009; Islam et al., 2018). In order to verify results, at least 20% of the isolates representative of each distinct haplotype were subjected to three independent analyses.

Multilocus SSR haplotypes were identified using HAPLOTYPE-ANALYSIS V 1.05 (Eliades and Eliades, 2009). Prior to genetic analyses, sample correction was performed in order to include each unique haplotype only once for each sample. Allelic and genetic diversity, including the number of alleles, number of private alleles (alleles detected only either in the L- or S-morphotype), and haploid genetic diversity (H) were calculated using GenAlEx version 6.51b2 (Peakall and Smouse, 2006; Smouse et al., 2017). In order to compare allelic diversity between *A. flavus* S and L morphotypes, SSR data of 391 L-morphotype isolates was obtained from surveys performed by the USDA-ARS Aflatoxin Laboratory in Tucson, AZ. These isolates originated from soils in Arizona (113 isolates, collected from 1997 to 2000), maize in southeastern US (162 isolates, collected 2015 to 2016), and maize and soils in Texas (116 isolates, collected from 2017 to 2018).

### Aflatoxin Production in Maize

**FIGURE 2:**
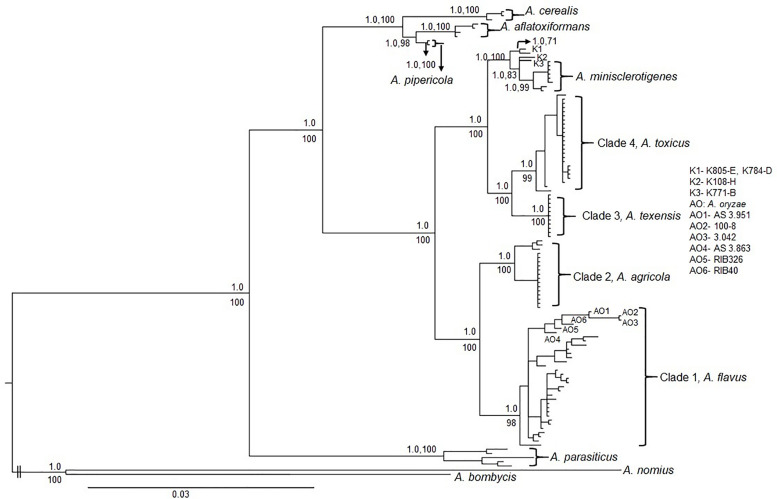
Mid-point rooted Bayesian phylogeny of S-morphology fungi and other aflatoxin producers within *Aspergillus* section *Flavi* based on concatenated partial sequence of *cmdA* (1.9 kb) and *niaD* (2.1kb). K1, K2 and K3 are lineages consisting of Kenyan fungi; K1: Isolates K805-E and K784-D, K2: Isolate K108-H and K3: Isolate K771-B (Probst et al., 2012). AO: *A. oryzae*; AO1: AS 3.951, AO2: 100-8, AO3: 3.042, AO4: AS 3.863, AO5: RIB326, and AO6: RIB40. Values above nodes or before commas are Bayesian posterior probabilities and values below nodes or after commas are maximum likelihood bootstrap support from 500 replicates.

Fungi were selected for aflatoxin assays in maize based on results from phylogenetic reconstruction. Four fungal isolates were selected from each phylogenetically distinct clade of S-morphology fungi detected within the US (Figure 2) by including isolates originating from different states (when possible) or different regions within the same state. Isolates were inoculated on sterile maize as described previously (Probst and Cotty, 2012). Briefly, healthy and undamaged maize kernels (Pioneer hybrid N82VGT) were increased to 25% water content and autoclaved in Erlenmeyer flasks (5 g per flask) for 20 min at 121°C. After sterilization, each flask was inoculated with 100 μl of conidial suspension adjusted to 10^6^ conidia ml^–1^. Maize was adjusted to 30% water content and incubated for 7 d at 25, 30, 35, and 40°C. Comparisons were made among clades at each temperature using the four distinct isolates per clade as replicates. Each experiment was performed twice.

Aflatoxins were extracted at the end of the incubation period by addition of 50 ml of 85% acetone. Maize-fungus cultures were ground to homogeneity in a laboratory grade Waring Blender (seven-speed laboratory blender, Waring Laboratory, Torrington, CT, United States) at full speed for 30 s and left in the dark for at least 1 h to allow fungal cell lysis and release of aflatoxins. The culture filtrates were separated on thin-layer chromatography (TLC) plates (Silica gel 60, EMD, Darmstadt, Germany) alongside aflatoxin standards (Aflatoxin Mix Kit-M, Supelco, Bellefonte, PA, United States) containing a mixture of known concentrations of aflatoxins B_1_, B_2_, G_1_, and G_2_. TLC plates were developed in ethyl ether-methanol-water (96:3:1) and air-dried, and aflatoxins were visualized under 365-nm UV light. Samples initially negative for aflatoxins were diluted with an equal volume of water and extracted twice with 25 ml of dichloromethane. Extracts were passed through a bed of anhydrous Na_2_SO_4_, combined, dried and resuspended in a volume of dichloromethane that allowed accurate quantification. Concentrated extracts were subjected to TLC as above. Aflatoxins were quantified with a scanning densitometer (TLC Scanner 3, Camag Scientific Inc., Wilmington, NC, United States).

### Taxonomy

Two previously undescribed taxa revealed by the phylogenetic analyses were characterized by incubating isolates for 7 d in the dark at 25, 30, 37, and 42°C following center point inoculation on Czapek agar, Czapek agar with 0.5% yeast extract (CYA), Malt extract agar (MEA, Difco), and V8 agar (5% V8 juice and 2% agar, pH = 6.0) according to Singh et al. (2018). Growth was evaluated by measuring colony diameters (four replicates per isolate) at each temperature.

All isolates were analyzed for production of aspergillic acid by inoculation onto *A. flavus* and *parasiticus* agar (AFPA; Pitt et al., 1983) and incubated for 7 d in the dark at 25, 30, and 37°C. Isolates were replicated four times at each temperature.

For micromorphological observations, mounts of 3 d old cultures grown on Czapek agar were made in lactophenol cotton blue with a drop of ethanol to wash excess conidia. Fungal structures were viewed and captured with a differential interference contrast Leica DMI6000B microscope (Leica Microsystems, Buffalo Grove, IL, United States) equipped with a Hamamatsu Flash 4.0 (Hamamatsu Corporation, Bridgewater, NJ, United States) digital camera and the software package Leica Application Suite LAS v 3.3.

Aflatoxin production by all isolates of each new taxon were evaluated as above by inoculating 100 μl of conidial suspensions (10^6^ conidia ml^–1^) on sterile maize (5 g flask^–1^) followed by incubation at 31°C for 7 d, after which aflatoxins were extracted and quantified.

The type and other representative isolates of both species described in the current study have been submitted to the ARS Culture Collection (NRRL) (Peoria, IL, United States) and the Fungal Genetics Stock Center, Manhattan, KS (Supplementary Table S1).

### Data Analysis

Total aflatoxins were measured in μg kg^–1^. Aflatoxin concentrations produced by each phylogenetic clade at individual temperatures were subjected to analysis of variance and Tukey’s HSD test (*p* = 0.05), as implemented in JMP 11.1.1 (SAS Institute, Cary, NC, United States, 2013). Fungal colony diameters were measured in millimeters (mm). Aflatoxin concentrations were tested for normality prior to statistical analyses and log-transformed if required. True means are presented for clarity.

## Results

### Phylogenetic Analyses

Phylogenetic reconstruction using individual and concatenated *cmdA* and *niaD* sequences resolved US S-morphology isolates into four distinct clades (Figure 2 and Supplementary Figures S1, S2). Both Bayesian inference and maximum likelihood analyses yielded similar topologies for individual and concatenated trees with high Bayesian posterior probabilities and bootstrap support for each of the four clades. The first clade consisted of B aflatoxin producers and was identified as *A. flavus* based on reference isolates (e.g., NRRL 3251, AF70, AF42, and AF12). *A. flavus* S-morphotype was the predominant S-morphology fungus detected in the US (Table 3). The *A. flavus* S-morphotype was found in all examined geographic regions, and was the only S-morphology species detected in Arizona (Table 3). The second clade is sister to *A. flavus* and included S-morphology isolates that also produce only B aflatoxins. Isolates in this clade originated from soils and maize and include five isolates previously reported as *A. flavus* S-morphotype. Two of these are from Texas [TXA35-K and TX06CB 9-G (NRRL 66873)] and three are from Thailand (Sukhothai19, Sanpatong22, and Ubon3). However, this clade was phylogenetically distinct from *A. flavus*, in both individual and concatenated phylogenies of partial sequences of *cmdA* and *niaD*, and is therefore delineated as a novel taxon, *Aspergillus agricola* (see “Taxonomy” below).

**TABLE 3:** Incidence of S-morphology fungi within *Aspergillus* section *Flavi* in the US.

**Region^a^**	**# of Isolates**	**Species^b^ (%)**			
		***A. flavus***	***A. texensis***	***A. toxicus***	***A. agricola***
Arizona	145	100	0	0	0
Southeastern US	68	78	3	19	0
Texas	281	88	3.2	5.3	4.3

*^*a*^Region of origin of isolates in the US. Southeastern US includes Alabama, Arkansas, Georgia, Louisiana and Mississippi. ^*b*^Assignment of fungal isolates to species based on molecular and phylogenetic analyses in the current study.*

The third clade, previously described as *A. texensis* (e.g., reference isolate NRRL 66855), consisted of B and G aflatoxin producers. Isolates belonging to *A. texensis* originated from Texas and the southeastern US. The fourth and final clade consisted of B aflatoxin producers that were recovered from maize and soil samples from the southeastern US and Texas. Several isolates of LAF (Probst et al., 2012) were included in the phylogenetic analysis, and these LAF isolates resolved into multiple lineages in both Bayesian and ML phylogenies (Figure 2), indicating they were a polyphyletic group. Five of these LAF isolates (two Kenyan isolates K44-K and K849-B, and three isolates from Texas TX07CB 73-I, TXLaFeria 2-F, and TX04A5-B), grouped with isolates in clade four. The remaining Kenyan LAF isolates resolved into multiple lineages, which were distinct from the fourth clade (Figure 2). It was therefore concluded that clade four represents a new taxon, described in the current study as *Aspergillus toxicus* (see “Taxonomy” below). *Aspergillus texensis* was sister to *A. toxicus*. Both *A. texensis* and *A. toxicus* were closely related to but distinct from *A. minisclerotigenes* (e.g., NRRL A-11611) and to the other Kenyan S isolates (e.g., K784-D, K805-E, K108-H and K771-B).

### Deletions in the *norB-cypA* Region of Aflatoxin Cluster

PCR amplification of the *norB-cypA* region of the aflatoxin biosynthesis cluster using primers from Ehrlich et al. (2004), and alignment with reference isolate *A. parasiticus* SU-1, revealed a 1.5 kb deletion in *A. flavus* S-morphotype isolates, a 0.9 kb deletion in *A. agricola*, and an intact *norB-cypA* region in *A. texensis*. Four isolates in clade four and both the Kenyan isolates did not yield an amplification product with primers from Ehrlich et al. (2004) but did amplify with primers reported by Probst et al. (2012) (Table 2). These amplicons revealed a 2.2 kb deletion. However, several isolates in clade four failed to yield amplification products with either set of primers, suggesting either variation in priming sites or structural variation distinct from other fungi that produce only B aflatoxins.

Based on both the phylogenetic analyses and deletions in the *norB*-*cypA* region of the aflatoxin biosynthesis cluster, 89.7% of the 494 US S-morphology isolates were assigned to the *A. flavus* S-morphotype, 5.7% to *A. toxicus*, 2.4% to *A. agricola*, and 2.2% to *A. texensis*.

### Aflatoxigenicity of S-morphology Species

Each S-morphology isolate from each of the four clades produced >500 μg kg^–1^ total aflatoxins at 25, 30, and 35°C in maize (Table 4). *Aspergillus texensis* (B_1_, B_2_, G_1_, and G_2_ aflatoxins), *A. agricola* (B_1_, B_2_ aflatoxins) and *A. toxicus* (B_1_, B_2_ aflatoxins) produced the highest concentrations of total aflatoxins at 30°C, whereas maximum total aflatoxin production by the *A. flavus* S-morphotype (B_1_, B_2_ aflatoxins) occurred at 35°C (Table 4). Aflatoxin production by S-morphology fungi from each clade was at least 90 times higher at 30°C, and 70 times higher at 35°C, compared to that at 25°C. The total concentrations of aflatoxins produced did not differ among species at 25°C (*p* = 0.302) or 30°C (*p* = 0.106). The *A. flavus* S-morphotype produced significantly higher concentrations of aflatoxins than *A. texensis*, *A. agricola*, and *A. toxicus* at 35°C (*p* < 0.01). Aflatoxin production was low (less than 100 μg kg^–1^ total aflatoxins) for *A. texensis, A. agricola*, and *A. toxicus* at 40°C, although *A. flavus* S isolates still produced significantly higher concentrations of total aflatoxins than the other three taxa at 40°C (Mean = 6,519 μg kg^–1^; range = 3,558–14,118 μg kg^–1^ total aflatoxins; *p* < 0.001).

**TABLE 4:** Aflatoxin production by four *Aspergillus* section *Flavi* species with S-morphology from the United States in maize.

**Species**	**Aflatoxins**	**Isolate**	**State, region, source**	**Total Aflatoxin (μg kg^–1^)**			
				**25°C**	**30°C**	**35°C**	**40°C**
*A. flavus* S-morphotype	B_1_, B_2_	AF70	AZ, Yuma, soil	1,849	313,371	530,896	4,119
		GNFHP4 C	AL, Morgan, maize	1,211	103,266	306,738	14,118
		E2-H	TX, San Patricio, soil	751	131,868	189,690	4,282
		JBABJB E	GA, Bryan, maize	797	103,421	399,681	3,558
		Mean		1,152	162,981	356,751A	6,519A
*A. Agricola*	B_1_, B_2_	NRRL 66869	TX, Coastal Bend, soil	1,021	141,992	67,155	72
		J55-H	TX, Gregory, soil	1,860	159,572	119,737	38
		NRRL 66872	TX, Bee, maize	944	37,092	31,649	45
		BC09-F	TX, Bexar, maize	1,492	174,615	166,208	241
		Mean		1,329	128,318	96,187B	99B
*A. texensis*	B_1_, B_2_, G_1_, G_2_	NRRL 66855	TX, Ellis, soil	664	262,663	72,274	41
		NRRL 66856	TX, Fort Bend, maize	1,225	191,641	74,453	16
		NRRL 66859	AR, Greene, maize	1,041	170,988	63,677	28
		J35-E	TX, San Patricio, soil	843	301,667	58,245	21
		Mean		943	231,740	67,162B	26B
*A. toxicus*	B_1_, B_2_	J15-B	TX, Gregory, soil	596	167,163	80,555	19
		CR20-D	TX, Coastal Bend, soil	1,375	246,556	95,029	24
		BRG3458 H	LA, Franklin, maize	542	190,850	186,866	33
		VC8-L	TX, Calhoun, maize	648	54,657	26,735	21
		Mean		790	164,807	97,296B	24B

*Total aflatoxin concentrations were compared between species at each temperature. Mean aflatoxin concentrations are averages of results from four isolates. Means followed by different upper-case letters within a column differ significantly according to Tukey’s HSD (*p* < 0.01). Means within a column lacking a letter do not differ (ANOVA, *p* > 0.05).*

### Genetic Diversity Within the *A. flavus* S-morphotype

Simple sequence repeat primers, previously designed for the L-morphotype of *A. flavus*, successfully amplified all 17 loci of *A. flavus* S-morphotype isolates (*n* = 443). Amplifications were free of PCR artifacts, and final primer combinations in multiplex PCRs generated only a single peak in the expected size range for each locus. There were no missing data or null alleles. *A. flavus* S-morphotype isolates were diverse, with 3–20 alleles per locus, 0.089–0.811 haploid gene diversity (Table 5), and separated into 202 haplotypes. The L-morphotype of *A. flavus* displayed greater diversity with a higher number of alleles per locus (7–38), haploid gene diversity of 0.525–0.908, and 257 haplotypes among 391 isolates. Eight haplotypes were shared between the two morphotypes, while several alleles were private to the L-morphotype (Table 5).

**TABLE 5:** Haploid diversity for 17 SSR loci of *Aspergillus flavus* S-morphotype isolates recovered from across the US during 2002–2017 compared to haploid diversity of the L-morphotype.

**PCR Panel**	**SSR Locus**	**Chromosome**	Repeat motif and Scaffold*	**S-morphotype**				**L-morphotype**			
				**No. of Alleles^**Φ**^**	**Size (bp)**	***h***	**No. of Private Alleles**	**No. of Alleles^**Φ**^**	**Size (bp)**	***h***	**No. of Private Alleles**
A	AF28	1L	(TTG)_11_/2504	11	116–152	0.516	1	12	113–148	0.525	2
	AF13	4U	(CTT)_9_/1866	18	122–182	0.757	2	19	122–200	0.856	3
	AF43	7U	(GAG)_13_/2634	12	367–408	0.795	0	20	365–438	0.864	8
	AF22	3L	(TTTA)_8_/2911	4	144–188	0.544	0	11	144–222	0.736	7
	AF31	6U	(TTC)_31_/2634	12	299–358	0.736	1	26	299–391	0.759	15
B	AF53	2L	(TCT)_8_/1918	5	131–154	0.527	0	11	128–163	0.634	6
	AF34	3L	(GTC)_4_(GTT)_8_/2911	5	296–314	0.657	0	14	290–403	0.780	9
	AF42	6U	(TTC)_16_/2634	19	143–296	0.539	4	28	139–250	0.865	13
	AF8	7L	(AAG)_16_/2911	19	147–218	0.811	2	26	144–236	0.838	9
D	AF16	5L	(TTG)_10_/2541	5	169–206	0.482	1	13	165–428	0.650	9
	AF54	2L	(ACAT)_8_/1918	3	157–165	0.239	0	7	157–180	0.647	4
	AF17	2L	(AGA)_4_(AGG)_10_/1918	10	347–385	0.624	0	16	344–388	0.818	6
	AF11	1L	(AAG)_12_/2504	10	126–228	0.686	1	27	117–215	0.848	18
E	AF66	4L	(AT)_12_/1569	7	259–275	0.691	1	13	253–286	0.508	7
	AF64	2U	(AC)_16_/2856	20	159–227	0.671	2	38	159–269	0.908	20
	AF63	2U	(AT)_7_/2856	5	125–135	0.089	0	7	125–137	0.576	2
	AF55	8U	(GT)_10_/1739	13	162–195	0.753	1	19	162–203	0.840	7
				**10.47^y^**				**18.06^x^**			

*^∗^Data from Grubisha and Cotty (2009). ΦNumber of alleles at each SSR locus in S and L morphotypes of *A. flavus*. Average number of alleles in each morphotype is indicated in bold and was significantly different between the two morphotypes (Student’s *t-*test, *p* < 0.01). Size – Range of allele sizes in base pairs (bp) at each SSR locus. *h*, Haploid gene diversity calculated using GenAlEx 6.51b2 (Peakall and Smouse, 2006; Smouse et al., 2017).*

Of the 202 *A. flavus* S-morphotype haplotypes identified, 16 were detected in more than one location, with three haplotypes, including the most frequent one, present in all regions sampled (Arizona, southeastern US, and Texas). Five haplotypes were shared only between Texas and Arizona, six only between Texas and the southeastern US, and two only between Arizona and the southeastern US. Seventy-four haplotypes were unique to Arizona (Table 6), of which 20 were displayed by two or more isolates. Out of 15 and 98 private haplotypes in the southeastern US and Texas, respectively, two haplotypes in the southeastern US and 28 in Texas were detected more than once (Table 6). Haploid genetic diversity varied among locations in an area-wide comparison, with the highest diversity in Texas, followed by the southeastern US and Arizona (Table 6).

**TABLE 6:** Genetic diversity of *Aspergillus flavus* S-morphotype isolates recovered from soil and maize samples across the US from 2002 to 2017.

**Region**	**N**	**N_S_**	**N_H_**	**N_PH_**	**N_A_ (range)**	**H_A_**
Arizona	145	145	84	74	5.118 (2-10)	0.505
Southeastern US^a^	54	38	25	14	5.412 (1-13)	0.577
Texas	245	206	112	98	8.588 (3-17)	0.612

*N, Total number of *A. flavus* S-morphotype isolates; N_*S*_, Number of isolates after sample correction; N_*H*_, Number of haplotypes; N_*PH*_, Number of private haplotypes (Haplotypes detected only in a single region); N_*A*_, Average number of alleles across 17 polymorphic SSR loci; H_*A*_, Haploid gene diversity calculated using GenAlex 6.51b2 (Smouse et al., 2017). ^*a*^Southeastern US includes Alabama, Arkansas, Georgia, Louisiana and Mississippi.*

### Taxonomy

**FIGURE 3:**
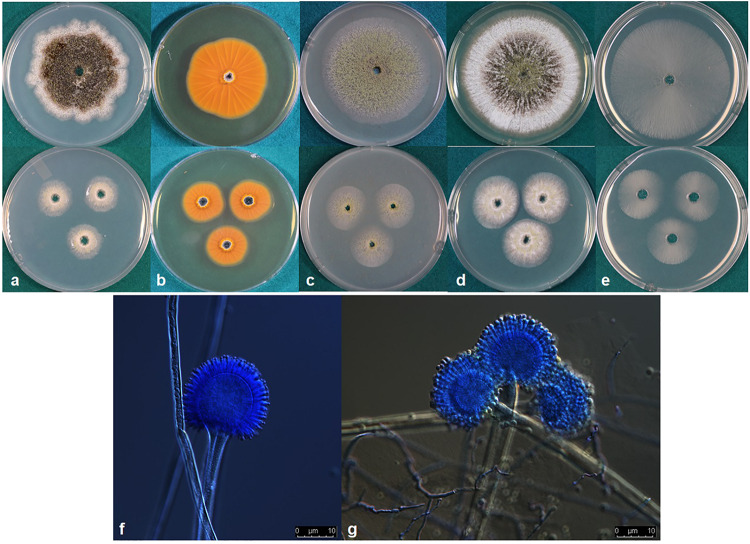
**(a–e)** Colonies of *Aspergillus agricola* (NRRL 66869) grown at 25°C for 7 d **(top)** and 3 d **(bottom)** on **(a)** Czapek, **(b)** reverse on AFPA, **(c)** V8 agar, **(d)** Czapek with yeast extract, and **(e)** MEA agar. **(f,g)** conidiophores.

***Aspergillus agricola*** P. Singh, K. A. Callicott, M. J. Orbach and P. J. Cotty **sp. nov.** MycoBank MB830377. (Figure 3).

*Etymology*: The species epithet refers to “soil inhabitant,” the substrate from which the type was isolated.

*Diagnosis*: *A. agricola* is closely related to *A. flavus*. *A. flavus* grows faster than *A. agricola* at 42°C on Czapek agar, CYA, MEA, and V8 agar. However, *A. agricola* grows faster than *A. flavus* at 25°C on MEA and V8. *A. agricola* produces visibly more conidia on CZ, CYA, and V8 than *A. flavus* S-morphotype isolates. The *A. flavus* S-morphotype contains a 1.5 kb deletion in the *norB-cypA* region of the aflatoxin biosynthesis gene cluster, whereas *A. agricola* contains a 0.9 kb deletion.

*Typus:* United States of America, Texas, Coastal Bend, soil cropped to maize (*Zea mays*), collected by P.J.Cotty (Holotype: NRRL 66869, ex-type: CR9-G = A2400).

*Colony* characteristics, 7 d: CYA at 25°C 69–74 mm, 76–77 mm at 30°C, 75–77 mm at 37°C, and 5–11 mm at 42°C. On CYA at 25°C: Moderately deep colony, smooth edges, conidia mainly around inoculation point, immature white sclerotia near edges but more mature black sclerotia near the colony center, reverse buff to dark brown. Czapek agar at 25°C 53–62 mm, 58–74 mm at 30°C, 58–68 mm at 37°C and 6–10 mm at 42°C. On Czapek agar at 25°C: Moderately deep colony with rough edges, conidia sparse, colony dominated by dark black sclerotia, immature white sclerotia at the edge. MEA agar at 25°C 59–64 mm, 75–77 mm at 30°C, 56–69 mm at 37°C and 3–7 mm at 42°C. On MEA agar at 25°C: Colony dominated by thin white mycelia, immature white sclerotia, conidia and a few dark sclerotia at and around inoculation point, colony edge smooth. V8 agar at 25°C 57–63 mm, 76–77 mm at 30°C, 66–71 mm at 37°C, and 3–6 mm at 42°C. On V8 agar at 25°C: Moderately deep colonies, dense conidia on entire colony surface, colony surface velvety, mature dark sclerotia toward the center, immature white sclerotia near the edge, colony edge smooth. AFPA at 25°C: Bright-orange reaction on the reverse side of AFPA medium indicating production of aspergillic acid (Figure 3b). Colony texture floccose, white mycelia, dark sclerotia at the inoculation point but mostly immature white sclerotia on the colony surface. Maximum radial growth of *A. agricola* occurred at 30°C on all media.

*Micromorphology:* Abundant production of dark black sclerotia on the agar surface was observed on Czapek, CYA, MEA, and V8 agar (Figure 3). Sclerotium size 150–350 μm. Fungal isolates produced light green conidia on all media tested; conidia circular and smooth walled, conidia diameter 2.5–5 μm, conidial heads uniseriate. Vesicle globose, 30–70 μm diameter. Conidiophores with smooth stipes, hyaline, 300–600 × 4–7 μm, phialides 6–8 × 2–4 μm.

*Aspergillus agricola* produced B_1_ and B_2_ aflatoxins in maize. Aflatoxin concentrations ranged from 96,434–648,171 μg kg^–1^ AFB_1_ (mean = 269,982 μg kg^–1^) and 1,860–23,951 μg kg^–1^ AFB_2_ (mean = 11,287 μg kg^–1^).

**FIGURE 4:**
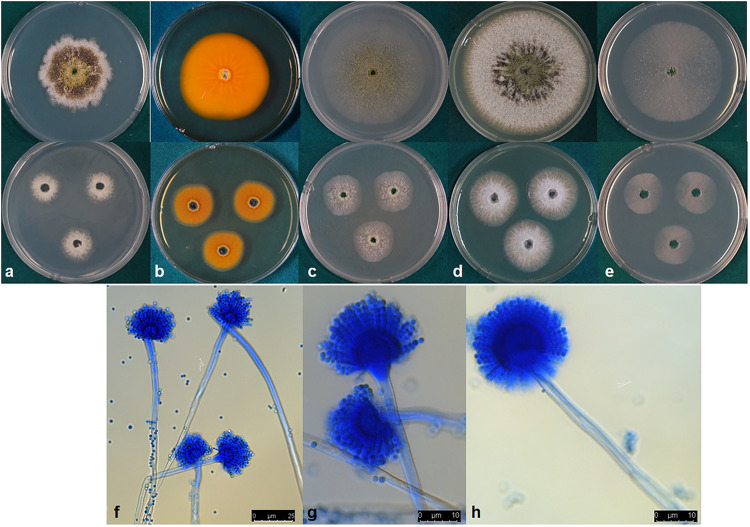
**(a–e)** Colonies of *Aspergillus toxicus* (NRRL 66898) grown at 25°C for 7 d **(top)** and 3 d **(bottom)** on **(a)** Czapek, **(b)** reverse on AFPA, **(c)** V8 agar, **(d)** Czapek with yeast extract, and **(e)** MEA agar. **(f–h)** conidiophores.

***Aspergillus toxicus***, P. Singh, K. A. Callicott, M. J. Orbach and P. J. Cotty **sp. nov.** MycoBank MB832486 (Figure 4).

*Etymology*: The species epithet refers to “toxic” because isolates of this species produce high concentrations of aflatoxins.

*Diagnosis*: *A. toxicus* is closely related to *A. texensis*. *A. toxicus* grows faster than *A. texensis* on Czapek and MEA agar at 25°C and V8 agar at 30°C. However, growth at 42°C on MEA and CYA is slower than that of *A. texensis*. *A. toxicus* produces aflatoxins B_1_ and B_2_ whereas *A. texensis* produces aflatoxins B_1_, B_2_, G_1_, and G_2_.

*Typus:* United States of America, Texas, Hidalgo, soil cropped to maize (*Zea mays*), collected by P.J.Cotty (Holotype: NRRL 66898; ex-type: A5-B-S = A2406).

*Colony characteristics*, 7 d: CYA at 25°C 63–73 mm, 76–77 mm at 30°C, 72–74 mm at 37°C, and 9–10 mm at 42°C. On CYA at 25°C: Moderately deep colony with smooth margins, conidia and mature black sclerotia mainly around inoculation point, majority of the colony dominated by immature white sclerotia, reverse buff to dark brown. Czapek agar at 25°C 42–56 mm, 61–69 mm at 30°C, 55–66 mm at 37°C and 5–7 mm at 42°C. On Czapek agar at 25°C: Moderately deep colony with rough margins, sparse conidia, colony mostly covered by dark black sclerotia, immature white sclerotia at the edge of the colony. MEA agar at 25°C 55–61 mm, 69–73 mm at 30°C, 69–72 mm at 37°C and 7–9 mm at 42°C. On MEA agar at 25°C: White, velvety colony mostly dominated by immature white sclerotia and thin white mycelia, dark sclerotia and conidia only near inoculation point. V8 agar at 25°C 54–61 mm, 69–73 mm at 30°C, 64–66 mm at 37°C and 8–9 mm 42°C. On V8 agar at 25°C: Moderately deep colony with velvety surface. Sparse conidia around inoculation point, colony dominated by sclerotia that are dark toward the center and white near the edges. On AFPA at 25°C: Bright-orange reaction on AFPA reverse indicating production of aspergillic acid, colony texture floccose, dense white mycelia, dark sclerotia near the inoculation point and immature white sclerotia elsewhere (Figure 4). Optimal growth occurred at 30°C on all media.

*Micromorphology:* Abundant production of dark black sclerotia on the agar surface was observed on Czapek, CYA, MEA, and V8 agar (Figure 4). Sclerotium size ranged from 100–300 μm. Conidia light yellowish-green; conidial heads uniseriate; conidia smooth circular; conidia diameter 2.4–4.6 μm; conidiophore stipes 450–800 × 6–11 μm smooth, hyaline; phialides 7–10.5 × 2–4 μm (Figure 4). Vesicle globose, 21–33 μm in diameter (Figure 4).

*Aspergillus toxicus* produced B_1_ and B_2_ aflatoxins in maize (Aflatoxin B_1_: range 127,094–338,275 μg kg^–1^; mean = 221,054 μg kg^–1^ and Aflatoxin B_2_: range 1,979–6,322 μg kg^–1^; mean = 3,618 μg kg^–1^).

## Discussion

The S-strain morphotype of *A. flavus* was initially reported in the US, where in some regions it comprises the majority of aflatoxin-producing fungi and is an important cause of aflatoxin contamination (Cotty, 1989; Orum et al., 1997; Cotty et al., 2008; Jaime-Garcia and Cotty, 2010). There have been many studies on the diversity and population genetics of the L-morphotype of *A. flavus* due to the importance of non-aflatoxigenic strains as biocontrol agents for the prevention of aflatoxin contamination. However, although diversity of S-morphology aflatoxin-producing species has been reported from Sub-Saharan Africa, there have been no previous studies on diversity among aflatoxin-producing S-morphology fungi in North America. In the current study, phylogenetic analyses were used to resolve four genetically distinct clades (Figure 2 and Supplementary Figures S1, S2) within a large collection of aflatoxin-producing fungi in the US with S-morphology. Each of the resolved clades are monophyletic with high Bayesian posterior probability, and bootstrap support, in a multigene phylogeny constructed with a total of 4.0 kb of concatenated sequence data from two unlinked genes, *cmdA* (1.9 kb) and *niaD* (2.1 kb). These analyses suggested four distinct, well-diverged species. The *A. flavus* S-morphotype was the dominant S-morphology species detected in the US, followed by *A. toxicus* sp. nov., *A. agricola* sp. nov., and *A. texensis* (Table 3). The *A. flavus* S-morphotype was detected in all three geographic areas analyzed (Arizona, Texas, and the southeastern US), indicating adaptation sufficient to allow success in diverse environments and niches. *A. agricola* was only detected in Texas while *A. texensis* and *A. toxicus* occurred in both Texas and the southeastern US. The underlying mechanisms behind these species distributions remain unclear but may be attributed to differences in environmental conditions (e.g., average daily temperature, humidity), cropping-system or soil characteristics (Schroeder and Boller, 1973; Orum et al., 1997; Horn and Dorner, 1998, 1999; Cotty and Jaime-Garcia, 2007).

Application of molecular phylogenetic analyses to the systematics of aflatoxin producers with S-morphology has resulted in identification of several previously uncharacterized taxa includin*g A. aflatoxiformans, A. minisclerotigenes, A. cerealis*, and *A. texensis* (Pildain et al., 2008; Singh et al., 2018; Frisvad et al., 2019). Each of these species produces both B and G aflatoxins. In the current study, both *A. agricola and A. toxicus* are resolved. Both of these new species produce only B aflatoxins like *A. flavus*, a species which, under current nomenclature, contains both L and S morphotypes (Figure 2). Failure of aflatoxin producers to produce G aflatoxins frequently results from deletions in the *norB*-*cypA* region of the aflatoxin biosynthesis cluster (Ehrlich et al., 2004). Several such deletions have been described (Ehrlich et al., 2004; Probst et al., 2012). Previous studies reported deletions of 0.9 and 1.5 kb in both the L and S morphotypes of *A. flavus* (Probst et al., 2012). The current study suggests that the presence of the 0.9 kb deletion in an S-morphotype fungus is characteristic of *A. agricola.* This is supported in the current study by identification of the 0.9 kb deletion in isolates previously assigned to the *A. flavus* S-morphotype but placed into *A. agricola* with phylogenetic analyses. These isolates originated from Texas [TXA35-K and TX06CB 9-G (NRRL 66873)] and Thailand (Sukhothai19, Sanpatong22, and Ubon3) (Probst et al., 2012). Assignment of S-morphology fungi with the 1.5 kb *norB-cypA* deletion to *A. flavus* is supported by the phylogenetic grouping of S-morphotype fungi with 202 SSR haplotypes collected in the current study from the US, as well as fungi from the Philippines (Supplementary Table S1). Thus, the current study supports rapid differentiation of *A. agricola* from *A. flavus* through simple PCR of the *norB-cypA* deletion. However, the *cmdA/niaD* concatenated tree is more complex for S-morphology fungi containing the 2.2 kb deletion, associated with lethal aflatoxicosis in Kenya (Probst et al., 2012). In the current study, fungi with the 2.2 kb deletion are paraphyletic with a highly supported divergence between fungi closely related to, but distinct from, *A. minisclerotigenes* and fungi forming clade 4, which is sister to *A. texensis* (Figure 2 and Supplementary Table S1). Isolates in clade 4 originate from Kenya as well as from Texas and Louisiana in the US, and are described in the current work as a new species, *A. toxicus*. The four isolates with a 2.2 kb *norB-cypA* deletion, but more closely related to *A. minisclerotigenes* than to either *A. texensis* or *A. toxicus*, are from Kenya (Figure 2). Evaluation of additional isolates is needed in order to resolve these Kenyan fungi into one or more taxa.

The four S-morphology species identified in the current study differed in geographic distribution. Thus, across the US, communities of aflatoxin-producing fungi vary widely in both proportions composed of S-morphology fungi and the specific S-morphology species present (Cotty, 1997; Jaime-Garcia and Cotty, 2006). Although *A. minisclerotigenes* has been sampled in South America, Sub-Saharan Africa, Australia, and Europe (Pildain et al., 2008; Soares et al., 2012; Probst et al., 2014; Singh and Cotty, 2019), this species was not detected in the US during the current study. *Aspergillus texensis*, which made up 2% of the S-morphology isolates examined, is currently only known to exist in the US (Singh et al., 2018). The divergence of S-morphology communities among West Africa, East Africa and the US is similar (Cotty and Cardwell, 1999; Probst et al., 2014). Geographic separations among species may reflect ancient isolation that allowed independent evolution of lineages. The soil-bound existence of S-morphology fungi may have allowed greater levels of geographic isolation that facilitated the speciation process, more than for species that use airborne conidia for dispersal. Once humans began to transport seed across the globe, internally borne sclerotial masses (Garber and Cotty, 1997) may have facilitated the movement of minor quantities of S-morphology species from regions of origin.

Aflatoxin production by the four S-morphology species from the US was similar at 25 and 30°C, but the *A. flavus* S-morphotype was the most toxic at warmer temperatures (35 and 40°C) (Table 4). Aflatoxin concentrations produced on maize at 25°C were sufficient to be fatal (CDC, 2004; Kamala et al., 2018), and each species produced more than 100,000 μg kg^–1^ total aflatoxins in maize at 30°C, with *A. texensis* producing the highest concentrations of aflatoxins. Drastic reductions in aflatoxin biosynthesis at temperatures above 32°C are frequently discussed and examined for insights into the regulation of aflatoxin biosynthesis (Schindler et al., 1967; Joffe and Lisker, 1969; O’Brian et al., 2007). However, in the current study, aflatoxin production at 35°C by all S-morphology fungi exceeded 50,000 μg kg^–1^, even though aflatoxin concentrations produced by *A. texensis, A. agricola* and *A. toxicus* at 35°C were lower than that at 30°C (Table 4). In fact, the *A. flavus* S-morphotype produced the highest concentrations of aflatoxins at 35°C, and even at 40°C, with more than 3,000 μg kg^–1^ total aflatoxins. This contrasts with previous studies unable to detect aflatoxins at or above 37°C (Schindler et al., 1967; Joffe and Lisker, 1969; O’Brian et al., 2007). Failure to detect aflatoxins in prior studies may be due to employed assessment methods for aflatoxin-producing ability, including production in submerged liquid cultures rather than on a susceptible host. Assaying aflatoxin-producing ability in host tissue may give more realistic assessments because aflatoxin-production in liquid fermentation media does not correlate with toxigenicity of isolates in a viable host (Probst and Cotty, 2012). Furthermore, high aflatoxigenicity at warmer temperatures can significantly impact food safety and food security worldwide due to rising global temperatures (Blankenship et al., 1984; Bircan et al., 2008; Battilani et al., 2016).

Simple sequence repeat markers, previously designed for population studies of the L-morphotype of *A. flavus*, were applied in the current study for the first time to *A. flavus* S-morphotype, and were found to be equally useful. Genetic diversity among *A. flavus* L isolates resident in soils and crops has primarily been assessed using vegetative compatibility analyses, which have revealed high VCG diversity within *A. flavus* L-morphotype populations from warm-agroecologies (Bayman and Cotty, 1991; Novas and Cabral, 2002; Probst et al., 2011; Picot et al., 2018). High genetic diversity was also detected by SSR analyses with 9–72 alleles per locus detected in 2,744 *A. flavus* L-morphotype isolates from Kenyan soils (Islam et al., 2018). The current study detected 7 to 38 alleles per locus in 391 L-morphotype isolates, but only 3–20 alleles in 443 S-morphotype isolates from the US (Table 5) utilizing the same SSR loci as Islam et al. (2018). Haploid gene diversity was also lower in the *A. flavus* S-morphotype. Differences in genetic diversity between the L and S morphotypes of *A. flavus* may be attributed to higher population size of the L isolates in crop and soil samples; several studies investigating diversity of fungi within section *Flavi* from crops or soils in a region have reported predominance of *A. flavus* L isolates, with low incidence of fungi with S-morphology (Cardwell and Cotty, 2002; Atehnkeng et al., 2008; Donner et al., 2009; Probst et al., 2014). Furthermore, production of much greater quantities of conidia by L-morphology fungi may facilitate dispersal over larger distances, thereby contributing to a greater effective population size. Region-wide haploid genetic diversity among *A. flavus* S isolates was the highest in Texas, where the largest number of isolates originated, followed by the southeastern US and Arizona. Out of the 202 haplotypes detected, most occurred only in a single region, with only three haplotypes shared across all regions. While all Texas *A. flavus* S isolates were recovered from maize and soils cropped to maize, and all the southeastern US isolates were recovered from maize, all isolates from Arizona were from soils cropped to cotton. Differences in temperature, soil properties, average precipitation and regionally cultivated crops may favor certain fungal genotypes over others (Donner et al., 2009; Jaime-Garcia and Cotty, 2010; Mehl et al., 2012; Mehl and Cotty, 2013). This is further strengthened by our discovery that only *A. flavus* S-morphotype isolates were detected in Arizona, while the sympatric occurrence of multiple distinct aflatoxigenic species with S-morphology was detected in Texas (four species) and the southeastern US (three species), regions with more variable environmental conditions and diverse cropping systems.

Aflatoxin contamination is highly heterogeneous across regions, between fields, within a field, and among portions of an individual plant (Cotty, 1994). Some of this heterogeneity may be explained by the incidence of aflatoxin producers with S-morphology (Probst et al., 2007; Cotty et al., 2008). Results from the current study suggest that under certain conditions aflatoxin-producing species with S-morphology vary in aflatoxin-producing potential, and that variation in frequencies of different species may contribute to aflatoxin heterogeneity. Furthermore, differential distributions and responses to temperature suggest adaptive divergence among the four S-morphology species examined in this study. Differences in the S-morphology species present may cause variation in the epidemiology of aflatoxin contamination. This is the first report of aflatoxin production at extreme temperatures such as 40°C; in the current study aflatoxin production in the host exceeded 3,000 μg kg^–1^ for all four *A. flavus* S isolates tested. The S-morphotype of *A. flavus* is most common in hot, dry areas of Arizona and Texas (Cotty, 1997; Orum et al., 1997; Bock et al., 2004; Jaime-Garcia and Cotty, 2006), suggesting adaptation to higher temperature may influence distribution. In regions where the S-morphotype of *A. flavus* infects crops, contamination may be expected to proceed even under extreme temperatures, including in mature crops before harvest, during on-farm storage in cotton modules, in bins, during transport, and in seed piles (Jaime-Garcia et al., 2013). Similarly, *A. aflatoxiformans, A. minisclerotigenes* and the fungi responsible for deadly aflatoxicoses in Kenya have S-morphology and are associated with aflatoxin contamination of crops in semi-arid and sub-humid regions of Sub-Saharan Africa (Cardwell and Cotty, 2002; Donner et al., 2009; Probst et al., 2014; Agbetiameh et al., 2018; Singh and Cotty, 2019). The occurrence and incidence of S-morphology fungi may become an increasing threat to food safety and security with the occurrence of warmer environments under climate change (Bock et al., 2004; Cotty and Jaime-Garcia, 2007), but aflatoxigenicity of each of the S-morphology species detected within the US (Table 4) suggests an already present potential to severely contaminate crops.

Three decades have passed since fungi with S-morphology were first found to be highly frequent in certain regions where these fungi are responsible for most of the fungal population’s aflatoxin-producing potential (Cotty, 1989, 1997; Cotty et al., 2008). The initial DNA based phylogeny (Egel et al., 1994) of these S-morphology fungi suggested divergence into several taxa, as did early physiological comparisons (Egel et al., 1994; Cotty and Cardwell, 1999). Eventually sufficient population sampling and DNA sequencing was performed to support delineation of distinct S-morphology species including *A. minisclerotigenes* (Pildain et al., 2008), *A. texensis* (Singh et al., 2018), and several others (Frisvad et al., 2019). The species described in the current manuscript are an extension of this process and were selected for the study due to their importance to crop contamination in North America (Cotty, 1989; Cotty et al., 2008). The systematics of these taxa may be further clarified with more extensive sampling and analysis of populations across the globe.

Identification and characterization of aflatoxigenic fungi facilitates the development of management procedures for prevention of aflatoxin contamination of crops. Relative frequencies of aflatoxigenic genotypes can vary among crops, regions, seasons and years, such that a continuously fluctuating assembly of genetically diverse aflatoxin producers may exist in fields/regions. The current study provides insights into the diversity and incidence of highly toxigenic S-morphology fungi from regions of the US that suffer from the perennial risk of aflatoxin contamination. Four phylogenetically distinct species with differences in aflatoxin profiles and *norB-cypA* deletions in the aflatoxin cluster were detected. Aflatoxin management using non-aflatoxigenic isolates of *A. flavus* must consider the occurrence of these genetically distinct S-morphology fungi in US soils and crops, and biocontrol active ingredients should be selected not just for adaptive traits, such as long-term persistence under harsh conditions in target regions but also to be effective against S-morphology fungi (Mehl et al., 2012; Probst et al., 2012; Bandyopadhyay et al., 2016).

## Data Availability Statement

The datasets generated for this study are available on request to the corresponding author.

## Author Contributions

PS and PC contributed to conception and design of the study. PS performed the experiments, analysis and wrote first draft of the manuscript. KC, MO, and PC validated the methodology and analysis and provided supervision. All authors contributed to manuscript revision, and read and approved the submitted version.

## Conflict of Interest

The authors declare that the research was conducted in the absence of any commercial or financial relationships that could be construed as a potential conflict of interest. The reviewer GM declared a shared affiliation, with no collaboration, with several of the authors, PC and KC, to the handling editor at the time of the review.

## Supplementary Material

FIGURE S1 Mid-point rooted Bayesian phylogeny of S-morphology fungi and other aflatoxin producers within *Aspergillus* section *Flavi* based on partial sequence of *cmdA* (1.9 kb). AO: *A. oryzae*; AO1- AS 3.951, AO2- 100-8, AO3- 3.042, AO4- AS 3.863, AO5- RIB326, AO6- RIB40. Values above nodes or before commas are Bayesian posterior probabilities and values below nodes or after commas are maximum likelihood bootstrap support from 500 replicates. Node values shown as hyphens indicate < 70% bootstrap support in ML analysis.

FIGURE S2 Mid-point rooted Bayesian phylogeny of S-morphology fungi and other aflatoxin producers within *Aspergillus* section *Flavi* based on partial sequence of *niaD* (2.1 kb). K1, K2 and K3 are lineages consisting of Kenyan fungi; K1: Isolates K805-E and K784-D, K2: Isolate K108-H and K3: Isolate K771-B (Probst et al., 2012). AO: *A. oryzae*; AO1- AS 3.951, AO2- 100-8, AO3- 3.042, AO4- AS 3.863, AO5- RIB326, AO6- RIB40. Values above nodes or before commas are Bayesian posterior probabilities and values below nodes or after commas are maximum likelihood bootstrap support from 500 replicates. Node values shown as hyphens indicate < 70% bootstrap support in ML analysis.

TABLE S1 *Aspergillus* section *Flavi* isolates used for phylogenetic reconstruction in the current study.

TABLE S2 Isolates used in the current study with GenBank accession numbers. Sequences recovered from GenBank are indicated in bold.
