# Supplementary figures and images for: Molecular Analysis of S-morphology Aflatoxin Producers From the United States Reveals Previously Unknown Diversity and Two New Taxa
Source: Front Microbiol. 2020 Jun 11;11:1236. doi: 10.3389/fmicb.2020.01236 (PMC7315800; doi:10.3389/fmicb.2020.01236)

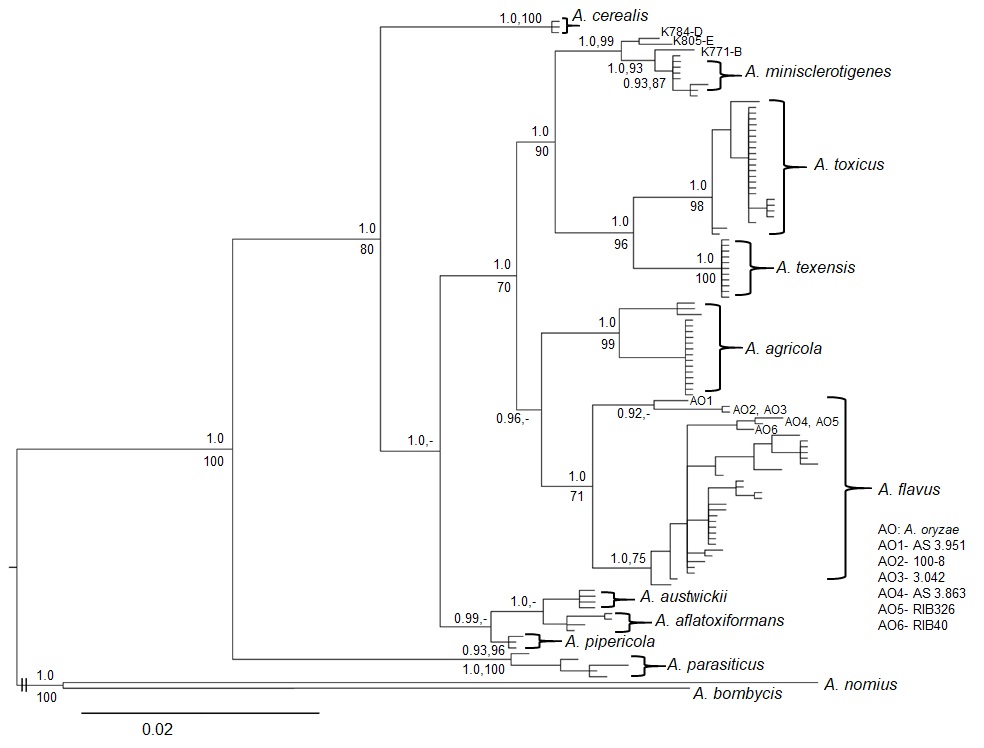

Supplement: FIGURE S1 — Mid-point rooted Bayesian phylogeny of S-morphology fungi and other aflatoxin producers within Aspergillus section Flavi based on partial sequence of cmdA (1.9 kb). AO: A. oryzae; AO1- AS 3.951, AO2- 100-8, AO3- 3.042, AO4- AS 3.863, AO5- RIB326, AO6- RIB40. Values above nodes or before commas are Bayesian posterior probabilities and values below nodes or after commas are maximum likelihood bootstrap support from 500 replicates. Node values shown as hyphens indicate < 70% bootstrap support in ML analysis. [file Image_1.JPEG]

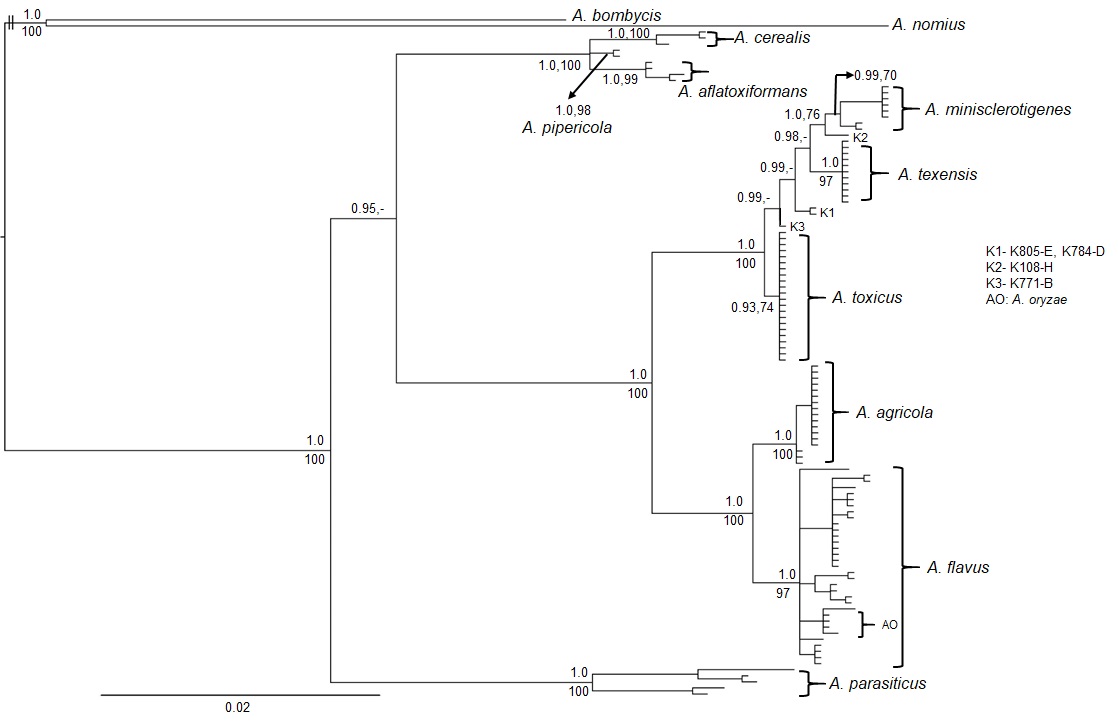

Supplement: FIGURE S2 — Mid-point rooted Bayesian phylogeny of S-morphology fungi and other aflatoxin producers within Aspergillus section Flavi based on partial sequence of niaD (2.1 kb). K1, K2 and K3 are lineages consisting of Kenyan fungi; K1: Isolates K805-E and K784-D, K2: Isolate K108-H and K3: Isolate K771-B (Probst et al., 2012). AO: A. oryzae; AO1- AS 3.951, AO2- 100-8, AO3- 3.042, AO4- AS 3.863, AO5- RIB326, AO6- RIB40. Values above nodes or before commas are Bayesian posterior probabilities and values below nodes or after commas are maximum likelihood bootstrap support from 500 replicates. Node values shown as hyphens indicate < 70% bootstrap support in ML analysis. [file Image_2.JPEG]
