# Supplementary material for: Molecular Analysis of S-morphology Aflatoxin Producers From the United States Reveals Previously Unknown Diversity and Two New Taxa
Source: Front Microbiol. 2020 Jun 11;11:1236. doi: 10.3389/fmicb.2020.01236 (PMC7315800; doi:10.3389/fmicb.2020.01236)
Supplement: TABLE S1 — Aspergillus section Flavi isolates used for phylogenetic reconstruction in the current study. [file Table_1.docx]

**Table S1** *Aspergillus* section *Flavi* isolates used for phylogenetic reconstruction in the current study.

| Isolate^a^ | Clade^b^ | Species | Source | Origin | AF^c^ | *norB-cypA* Deletion (kb) | Citation |
| --- | --- | --- | --- | --- | --- | --- | --- |
|  |  |  |  |  |  |  |  |
| NRRL 3251 | 1 | *A. flavus* | Walnut | California, USA | B | 1.5 | Hesseltine et al., 1970 |
| AF12 = ATCC^®^ MYA382 | 1 | *A. flavus* | Soil | Arizona, USA | B | 1.5 | Cotty, 1989 |
| AF42 = ATCC^®^ MYA383 | 1 | *A. flavus* | Cottonseed | Arizona, USA | B | 1.5 | Cotty, 1989 |
| AF70 = ATCC^®^ MYA384 | 1 | *A. flavus* | Soil | Arizona, USA | B | 1.5 | Cotty, 1989 |
| L1A3 | 1 | *A. flavus* | Soil | Philippines | B | 1.5 | Probst et al., 2012, Current Study |
| L1D2 | 1 | *A. flavus* | Soil | Philippines | B | 1.5 | Probst et al., 2012, Current Study |
| L2E1 | 1 | *A. flavus* | Soil | Philippines | B | 1.5 | Probst et al., 2012,  Current Study |
| MINIC4 | 1 | *A. flavus* | Soil | Philippines | B | 1.5 | Probst et al., 2012,  Current Study |
| V2D2 | 1 | *A. flavus* | Soil | Philippines | B | 1.5 | Probst et al., 2012,  Current Study |
| 31520-2405-2-H | 1 | *A. flavus* | Soil | Arizona, USA | B | 1.5 | Current Study |
| 30609-1803 SW-5-K | 1 | *A. flavus* | Soil | Arizona, USA | B | 1.5 | Current Study |
| GNFHP4 C | 1 | *A. flavus* | Maize | Alabama, USA | B | 1.5 | Current Study |
| VCSPWS H | 1 | *A. flavus* | Maize | Arkansas, USA | B | 1.5 | Current Study |
| AGSTNW E | 1 | *A. flavus* | Maize | Georgia, USA | B | 1.5 | Current Study |
| VPE01D G | 1 | *A. flavus* | Maize | Mississippi, USA | B | 1.5 | Current Study |
| BA12-J | 1 | *A. flavus* | Maize | Texas, USA | B | 1.5 | Current Study |
| WX13 C1-G | 1 | *A. flavus* | Maize | Texas, USA | B | 1.5 | Current Study |
| WX13 MX1-4B-J | 1 | *A. flavus* | Maize | Texas, USA | B | 1.5 | Current Study |
| A10-A-S | 1 | *A. flavus* | Maize | Texas, USA | B | 1.5 | Current Study |
| E28-L | 1 | *A. flavus* | Maize | Texas, USA | B | 1.5 | Current Study |
| J12-E | 1 | *A. flavus* | Maize | Texas, USA | B | 1.5 | Current Study |
| BC31-E | 1 | *A. flavus* | Maize | Texas, USA | B | 1.5 | Current Study |
| AT52-K | 1 | *A. flavus* | Maize | Texas, USA | B | 1.5 | Current Study |
| AF13 = ATCC^®^ 96044 | 1 | *A. flavus* | Soil | USA | B | 0.9 | Cotty, 1989 |
| CHL159 | 1 | *A. flavus* | Chili | Unknown | B | Unknown | Singh and Cotty, 2019 |
| CHL187 | 1 | *A. flavus* | Chili | Pakistan | B | Unknown | Singh and Cotty, 2019 |
| NRRL 3357 | 1 | *A. flavus* | Groundnut | USA | B | 0.9 | Ehrlich et al., 2004, NRRL Database |
| AS 3.951 | 1 | *A. oryzae* | Unknown | Unknown | None | 1.5 | NCBI (Genome sequenced |
| 100-8 | 1 | *A. oryzae* | Unknown | Unknown | None | 1.5 | NCBI (Genome sequenced |
| 3.042 | 1 | *A. oryzae* | Unknown | Unknown | None | 1.5 | NCBI (Genome sequenced |
| AS 3.863 | 1 | *A. oryzae* | Unknown | Unknown | None | 1.5 | NCBI (Genome sequenced |
| RIB326 | 1 | *A. oryzae* | Shoyu koji | Japan | None | 1.5 | Chang et al., 2006, Umemura et al., 2012 |
| RIB40 | 1 | *A. oryzae* | Cereal grain | Japan | None | 1.5 | NRRL Database, Chang et al., 2006 |
| NRRL 66869/ A2400 | 2 | *A. agricola* | Soil | Texas, USA | B | 0.9 | Current Study |
| NRRL 66870/ A2401 | 2 | *A. agricola* | Soil | Texas, USA | B | 0.9 | Current Study |
| NRRL 66871/ A2402 | 2 | *A. agricola* | Soil | Texas, USA | B | 0.9 | Current Study |
| NRRL 66872/ A2403 | 2 | *A. agricola* | Maize | Texas, USA | B | 0.9 | Current Study |
| NRRL 66873/ A2404/ TX06CB 9-G | 2 | *A. agricola* | Maize | Texas, USA | B | 0.9 | Probst et al., 2012 |
| TXA35-K | 2 | *A. agricola* | Soil | Texas, USA | B | 0.9 | Probst et al., 2012 |
| C3-J | 2 | *A. agricola* | Soil | Texas, USA | B | 0.9 | Current Study |
| A2-A | 2 | *A. agricola* | Soil | Texas, USA | B | 0.9 | Current Study |
| J15-H | 2 | *A. agricola* | Soil | Texas, USA | B | 0.9 | Current Study |
| J11-B | 2 | *A. agricola* | Soil | Texas, USA | B | 0.9 | Current Study |
| J11-C | 2 | *A. agricola* | Soil | Texas, USA | B | 0.9 | Current Study |
| E13-L | 2 | *A. agricola* | Soil | Texas, USA | B | 0.9 | Current Study |
| BC09-F | 2 | *A. agricola* | Maize | Texas, USA | B | 0.9 | Current Study |
| EC37-C | 2 | *A. agricola* | Maize | Texas, USA | B | 0.9 | Current Study |
| Sukhothai19 | 2 | *A. agricola* | Soil | Thailand | B | 0.9 | Ehrlich et al., 2007, Probst et al., 2012 |
| Sanpatong22 | 2 | *A. agricola* | Soil | Thailand | B | 0.9 | Probst et al., 2012 |
| Ubon3 | 2 | *A. agricola* | Soil | Thailand | B | 0.9 | Probst et al., 2012 |
| NRRL 66855 | 3 | *A. texensis* | Soil | Texas, USA | B,G | 0.0 | Singh et al., 2018 |
| NRRL 66856 | 3 | *A. texensis* | Maize | Texas, USA | B,G | 0.0 | Singh et al., 2018 |
| NRRL 66857 | 3 | *A. texensis* | Maize | Louisiana, USA | B,G | 0.0 | Singh et al., 2018 |
| NRRL 66858 | 3 | *A. texensis* | Soil | Texas, USA | B,G | 0.0 | Singh et al., 2018 |
| NRRL 66859 | 3 | *A. texensis* | Maize | Arkansas, USA | B,G | 0.0 | Singh et al., 2018 |
| J35-E | 3 | *A. texensis* | Soil | Texas, USA | B,G | 0.0 | Singh et al., 2018 |
| VC16-A | 3 | *A. texensis* | Soil | Texas, USA | B,G | 0.0 | Singh et al., 2018 |
| CTL-1I | 3 | *A. texensis* | Soil | Texas, USA | B,G | 0.0 | Singh et al., 2018 |
| P2R2-A Q | 3 | *A. texensis* | Soil | Texas, USA | B,G | 0.0 | Singh et al., 2018 |
| 1-1-O | 3 | *A. texensis* | Soil | Texas, USA | B,G | 0.0 | Singh et al., 2018 |
| 1-1L | 3 | *A. texensis* | Soil | Texas, USA | B,G | 0.0 | Singh et al., 2018 |
| K805-E = A1170 | LAF, K1 | N/A | Maize | Kenya | B | 2.2 | Probst et al., 2007 |
| K784-D = A1168 | LAF, K1 | N/A | Maize | Kenya | B | 2.2 | Probst et al., 2007 |
| K108-H | LAF, K2 | N/A | Maize | Kenya | B | 2.2 | Probst et al., 2012 |
| K771-B | LAF, K3 | N/A | Maize | Kenya | B | 2.2 | Probst et al., 2012 |
| K44-K | 4 | *A. toxicus* | Maize | Kenya | B | 2.2 | Probst et al., 2012,  Current Study |
| K849-B = A1171 | 4 | *A. toxicus* | Maize | Kenya | B | 2.2 | Probst et al., 2007,  Current Study |
| TX07CB73-I | 4 | *A. toxicus* | Maize | Texas, USA | B | Unknown | Probst et al., 2012, Current Study |
| TXLaFeria 2-F | 4 | *A. toxicus* | Cottonseed | Texas, USA | B | Unknown | Probst et al., 2012, Current Study |
| TX04A5-B | 4 | *A. toxicus* | Soil | Texas, USA | B | Unknown | Probst et al., 2012, Current Study |
| BRG3458 A | 4 | *A. toxicus* | Maize | Louisiana, USA | B | Unknown | Current Study |
| NRRL 66897/ A2405 | 4 | *A. toxicus* | Maize | Louisiana, USA | B | Unknown | Current Study |
| NRRL 66898/ A2406/ A5-B-S | 4 | *A. toxicus* | Soil | Texas, USA | B | Unknown | Probst et al., 2012, Current Study |
| NRRL 66899/ A2407 | 4 | *A. toxicus* | Soil | Texas, USA | B | Unknown | Current Study |
| NRRL 66900/ A2408 | 4 | *A. toxicus* | Maize | Texas, USA | B | Unknown | Current Study |
| BRG3458 H | 4 | *A. toxicus* | Maize | Louisiana, USA | B | Unknown | Current Study |
| BRG3458 J | 4 | *A. toxicus* | Maize | Louisiana, USA | B | Unknown | Current Study |
| BRG5138 J | 4 | *A. toxicus* | Maize | Louisiana, USA | B | Unknown | Current Study |
| CR20-D | 4 | *A. toxicus* | Soil | Texas, USA | B | Unknown | Current Study |
| D16-J | 4 | *A. toxicus* | Soil | Texas, USA | B | Unknown | Current Study |
| D25-A-S | 4 | *A. toxicus* | Soil | Texas, USA | B | Unknown | Current Study |
| E21-B | 4 | *A. toxicus* | Soil | Texas, USA | B | Unknown | Current Study |
| A34-N | 4 | *A. toxicus* | Soil | Texas, USA | B | Unknown | Current Study |
| CR24-F | 4 | *A. toxicus* | Soil | Texas, USA | B | Unknown | Current Study |
| J15-B | 4 | *A. toxicus* | Soil | Texas, USA | B | 2.2 | Current Study |
| CR10-G | 4 | *A. toxicus* | Soil | Texas, USA | B | 2.2 | Current Study |
| EC24-C | 4 | *A. toxicus* | Maize | Texas, USA | B | Unknown | Current Study |
| EC49-L | 4 | *A. toxicus* | Maize | Texas, USA | B | 2.2 | Current Study |
| BG14-F | 4 | *A. toxicus* | Maize | Texas, USA | B | 2.2 | Current Study |
| NRRL A-11612 | AA | *A. aflatoxiformans* | Groundnut | Nigeria | B,G | 0.0 | Hesseltine et al., 1970 |
| BN008R =  ATCC^®^MYA379 | AA | *A. aflatoxiformans* | Soil | Benin | B,G | 0.0 | Cotty and Cardwell, 1999 |
| BN038G =  ATCC^®^MYA380 | AA | *A. aflatoxiformans* | Soil | Benin | B,G | 0.0 | Cotty and Cardwell, 1999 |
| BN040B =  ATCC^®^MYA381 | AA | *A. aflatoxiformans* | Soil | Benin | B,G | 0.0 | Cotty and Cardwell, 1999 |
| BN009-E | AP | *A. parasiticus* | Soil | Benin | B,G | 0.0 | Probst et al., 2014 |
| NRRL 2999 | AP | *A. parasiticus* | Groundnut | Uganda | B,G | 0.0 | Rambo et al., 1974 |
| NRRL 465 | AP | *A. parasiticus* |  | USA | B,G | 0.0 | Wei and Jong, 1986 |
| NRRL 29538 | AP | *A. parasiticus* | Soil | USA | B,G | 0.0 | Peanut Lab, Dawson, GA (NRRL database) |
| NRRL 29590 | AP | *A. parasiticus* | Soil | USA | B,G | 0.0 | Peanut Lab, Dawson, GA (NRRL database) |
| NRRL A-11611 | AM | *A. minisclerotigenes* | Groundnut | Nigeria | B,G | 0.0 | Hesseltine et al. 1970 |
| TAR3N43 | AM | *A. minisclerotigenes* | Groundnut | Argentina | B,G | 0.0 | Probst et al., 2012 |
| 4-2 | AM | *A. minisclerotigenes* | Soil | Australia | B,G | 0.0 | Geiser et al., 1998 |
| CHL583 | AM | *A. minisclerotigenes* | Chili | Nigeria | B,G | 0.0 | Singh and Cotty, 2019 |
| CHL663 | AM | *A. minisclerotigenes* | Chili | Nigeria | B,G | 0.0 | Singh and Cotty, 2019 |
| CHL707 | AM | *A. minisclerotigenes* | Chili | Nigeria | B,G | 0.0 | Singh and Cotty, 2019 |
| CHL845 | AM | *A. minisclerotigenes* | Chili | Nigeria | B,G | 0.0 | Singh and Cotty, 2019 |
| CHL895 | AM | *A. minisclerotigenes* | Chili | Nigeria | B,G | 0.0 | Singh and Cotty, 2019 |
| NRRL 66708 | AC | *A. cerealis* | Groundnut | Côte d’Ivoire | B,G | 0.0 | Carvajal-Campos et al., 2017; Frisvad et al., 2019 |
| NRRL 66709 | AC | *A. cerealis* | Groundnut | Côte d’Ivoire | B,G | 0.0 | Carvajal-Campos et al., 2017; Frisvad et al., 2019 |
| NRRL 66710 | AC | *A. cerealis* | Groundnut | Côte d’Ivoire | B,G | 0.0 | Carvajal-Campos et al., 2017; Frisvad et al., 2019 |
| DTO 228-F7 | AA | *A. austwickii* | Rice | Nigeria | B,G | 0.0 | Frisvad et al., 2019 |
| DTO 228-F8 | AA | *A. austwickii* | Rice | Nigeria | B,G | 0.0 | Frisvad et al., 2019 |
| DTO 228-F9 | AA | *A. austwickii* | Rice | Nigeria | B,G | 0.0 | Frisvad et al., 2019 |
| DTO 228-G8 | AA | *A. austwickii* | Sesame | Nigeria | B,G | 0.0 | Frisvad et al., 2019 |
| DTO 228-H4 | AP | *A. pipericola* | Black pepper | Unknown | B,G | 0.0 | Frisvad et al., 2019 |
| CHL832 | AP | *A. pipericola* | Chili | Nigeria | B,G | 0.0 | Singh and Cotty, 2019 |
| CHL888 | AP | *A. pipericola* | Chili | Nigeria | B,G | 0.0 | Singh and Cotty, 2019 |
| NRRL 13137 | AN | *A. nomius* | Wheat | USA | B,G | 0.0 | Kurtzman et al., 1987 |
| NRRL 26010 | AB | *A. bombycis* | Frass, Silkworm | Japan | B,G | 0.0 | Peterson et al., 2001 |
|  |  |  |  |  |  |  |  |

^a^Fungal isolates with S-morphology from the current study and reference isolates from previously described aflatoxin-producing species.

^b^Clade number indicates phylogenetic clades identified in Figure 2. AA- *A. aflatoxiformans,* AC- *A. cerealis*, AM- *A. minisclerotigenes,* AP- *A. parasiticus,* AN- *A. nomius,* AB- *A. bombycis*, LAF- unnamed Lethal Aflatoxicosis Fungus from Kenya.

^c^Aflatoxin profiles of isolates. B- Production of B aflatoxins only and BG- Production of both B and G aflatoxins.
