# Supplementary material for: Molecular Analysis of S-morphology Aflatoxin Producers From the United States Reveals Previously Unknown Diversity and Two New Taxa
Source: Front Microbiol. 2020 Jun 11;11:1236. doi: 10.3389/fmicb.2020.01236 (PMC7315800; doi:10.3389/fmicb.2020.01236)
Supplement: TABLE S2 — Isolates used in the current study with GenBank accession numbers. Sequences recovered from GenBank are indicated in bold. [file Table_2.docx]

**Table S2** Isolates used in the current study with GenBank accession numbers. Sequences recovered from GenBank are indicated in bold.

| **Species** | **Isolate** | **GenBank Accession Number** | |
| --- | --- | --- | --- |
|  |  | ***cmdA*** | ***niaD*** |
| *A. flavus* | NRRL 3251 | MN987027 | **MH760534** |
| *A. flavus* | AF12 = ATCC^®^ MYA382 | MN987028 | **MH760531** |
| *A. flavus* | AF42 = ATCC^®^ MYA383 | MN987029 | **MH760532** |
| *A. flavus* | AF70 = ATCC^®^ MYA384 | MN987030 | **MH760533** |
| *A. flavus* | L1A3 | MN987031 | MN987129 |
| *A. flavus* | L1D2 | MN987032 | MN987130 |
| *A. flavus* | L2E1 | MN987033 | MN987131 |
| *A. flavus* | MINIC4 | MN987034 | MN987132 |
| *A. flavus* | V2D2 | MN987035 | MN987133 |
| *A. flavus* | 31520-2405-2-H | MN987036 | MN987134 |
| *A. flavus* | 30609-1803 SW-5-K | MN987037 | MN987135 |
| *A. flavus* | GNFHP4 C | MN987038 | MN987136 |
| *A. flavus* | VCSPWS H | MN987039 | MN987137 |
| *A. flavus* | AGSTNW E | MN987040 | MN987138 |
| *A. flavus* | VPE01D G | MN987041 | MN987139 |
| *A. flavus* | BA12-J | MN987042 | MN987140 |
| *A. flavus* | WX13 C1-G | MN987043 | MN987141 |
| *A. flavus* | WX13 MX1-4B-J | MN987044 | MN987142 |
| *A. flavus* | A10-A-S | MN987045 | MN987143 |
| *A. flavus* | E28-L | MN987046 | MN987144 |
| *A. flavus* | J12-E | MN987047 | MN987145 |
| *A. flavus* | BC31-E | MN987048 | MN987146 |
| *A. flavus* | AT52-K | MN987049 | MN987147 |
| *A. flavus* L-morphotype | AF13 = ATCC^®^ 96044 | MN987050 | **MH760530** |
| *A. flavus* L-morphotype | CHL159 | MN987051 | **MH760528** |
| *A. flavus* L-morphotype | CHL187 | MN987052 | **MH760529** |
| *A. flavus* L-morphotype | NRRL 3357 | **AAIH02000003** | **AAIH02000071** |
| *A. oryzae* | AS 3.951 | **AKXN01000961** | **AKXN01000525** |
| *A. oryzae* | 100-8 | **AMCJ01000129** | **AMCJ01000120** |
| *A. oryzae* | 3.042 | **AKHY01000168** | **AKHY01000133** |
| *A. oryzae* | AS 3.863 | **AKXL01000726** | **AKXL01000388** |
| *A. oryzae* | RIB326 | **BAEZ01000010** | **BAEZ01000013** |
| *A. oryzae* | RIB40 | **JZJM01000088** | **JZJM01000109** |
| *A. agricola* | NRRL 66869 | MN987053 | MN987148 |
| *A. agricola* | NRRL 66870 | MN987054 | MN987149 |
| *A. agricola* | NRRL 66871 | MN987055 | MN987150 |
| *A. agricola* | NRRL 66872 | MN987056 | MN987151 |
| *A. agricola* | NRRL 66873/ TX06CB 9-G | MN987057 | MN987152 |
| *A. agricola* | TXA35-K | MN987058 | MN987153 |
| *A. agricola* | C3-J | MN987059 | MN987154 |
| *A. agricola* | A2-A | MN987060 | MN987155 |
| *A. agricola* | J15-H | MN987061 | MN987156 |
| *A. agricola* | J11-B | MN987062 | MN987157 |
| *A. agricola* | J11-C | MN987063 | MN987158 |
| *A. agricola* | E13-L | MN987064 | MN987159 |
| *A. agricola* | BC09-F | MN987065 | MN987160 |
| *A. agricola* | EC37-C | MN987066 | MN987161 |
| *A. agricola* | Sukhothai19 | MN987067 | MN987162 |
| *A. agricola* | Sanpatong22 | MN987068 | MN987163 |
| *A. agricola* | Ubon3 | MN987069 | MN987164 |
| *A. texensis* | NRRL 66855 | MN987070 | **MK119684** |
| *A. texensis* | NRRL 66856 | MN987071 | **MK119685** |
| *A. texensis* | NRRL 66857 | MN987072 | **MK119686** |
| *A. texensis* | NRRL 66858 | MN987073 | **MK119687** |
| *A. texensis* | NRRL 66859 | MN987074 | **MK119688** |
| *A. texensis* | J35-E | MN987075 | MN987165 |
| *A. texensis* | VC16-A | MN987076 | MN987166 |
| *A. texensis* | CTL-1I | MN987077 | MN987167 |
| *A. texensis* | P2R2-A Q | MN987078 | MN987168 |
| *A. texensis* | 1-1-O | MN987079 | MN987169 |
| *A. texensis* | 1-1L | MN987080 | MN987170 |
| Lethal Aflatoxicosis Fungus, K1 | K805-E = A1170 | MN987081 | **MK119683** |
| Lethal Aflatoxicosis Fungus, K1 | K784-D = A1168 | MN987082 | **MK119682** |
| Lethal Aflatoxicosis Fungus, K2 | K108-H | MN987083 | MN987171 |
| Lethal Aflatoxicosis Fungus, K3 | K771-B | MN987084 | MN987172 |
| *A. toxicus* | K44-K | MN987085 | MN987173 |
| *A. toxicus* | K849-B = A1171 | MN987086 | MN987174 |
| *A. toxicus* | TX07CB73-I | MN987087 | MN987175 |
| *A. toxicus* | TXLaFeria 2-F | MN987088 | MN987176 |
| *A. toxicus* | TX04A5-B | MN987089 | MN987177 |
| *A. toxicus* | BRG3458 A | MN987090 | MN987178 |
| *A. toxicus* | NRRL 66897 | MN987091 | MN987179 |
| *A. toxicus* | NRRL 66898/A5-B-S | MN987092 | MN987180 |
| *A. toxicus* | NRRL 66899 | MN987093 | MN987181 |
| *A. toxicus* | NRRL 66900 | MN987094 | MN987182 |
| *A. toxicus* | BRG3458 H | MN987095 | MN987183 |
| *A. toxicus* | BRG3458 J | MN987096 | MN987184 |
| *A. toxicus* | BRG5138 J | MN987097 | MN987185 |
| *A. toxicus* | CR20-D | MN987098 | MN987186 |
| *A. toxicus* | D16-J | MN987099 | MN987187 |
| *A. toxicus* | D25-A-S | MN987100 | MN987188 |
| *A. toxicus* | E21-B | MN987101 | MN987189 |
| *A. toxicus* | A34-N | MN987102 | MN987190 |
| *A. toxicus* | CR24-F | MN987103 | MN987191 |
| *A. toxicus* | J15-B | MN987104 | MN987192 |
| *A. toxicus* | CR10-G | MN987105 | MN987193 |
| *A. toxicus* | EC24-C | MN987106 | MN987194 |
| *A. toxicus* | EC49-L | MN987107 | MN987195 |
| *A. toxicus* | BG14-F | MN987108 | MN987196 |
| *A. aflatoxiformans* | NRRL A-11612 | MN987109 | **MK119678** |
| *A. aflatoxiformans* | BN008R = ATCC^®^MYA379 | MN987110 | **MK119681** |
| *A. aflatoxiformans* | BN038G = ATCC^®^MYA380 | MN987111 | **MK119679** |
| *A. aflatoxiformans* | BN040B = ATCC^®^MYA381 | MN987112 | **MK119680** |
| *A. parasiticus* | BN009-E | MN987113 | **MH760538** |
| *A. parasiticus* | NRRL 2999 | MN987114 | **MH760537** |
| *A. parasiticus* | NRRL 465 | MN987115 | **MK119672** |
| *A. parasiticus* | NRRL 29538 | MN987116 | **MK119673** |
| *A. parasiticus* | NRRL 29590 | MN987117 | **MK119674** |
| *A. minisclerotigenes* | NRRL A-11611 | MN987118 | **MH760525** |
| *A. minisclerotigenes* | TAR3N43 | MN987119 | **MH760520** |
| *A. minisclerotigenes* | 4-2 | MN987120 | **MH760522** |
| *A. minisclerotigenes* | CHL583 | MN987121 | MN987197 |
| *A. minisclerotigenes* | CHL663 | MN987122 | MN987198 |
| *A. minisclerotigenes* | CHL707 | MN987123 | MN987199 |
| *A. minisclerotigenes* | CHL845 | MN987124 | MH760524 |
| *A. minisclerotigenes* | CHL895 | MN987125 | MN987200 |
| *A. cerealis* | NRRL 66708 | MN987126 | **MK119675** |
| *A. cerealis* | NRRL 66709 | MN987127 | **MK119676** |
| *A. cerealis* | NRRL 66710 | MN987128 | **MK119677** |
| *A. austwickii* | DTO 228-F7 | **MG518072** | Not Available |
| *A. austwickii* | DTO 228-F8 | **MG518073** | Not Available |
| *A. austwickii* | DTO 228-F9 | **MG518074** | Not Available |
| *A. austwickii* | DTO 228-G8 | **MG518082** | Not Available |
| *A. pipericola* | DTO 228-H4 | **MG518087** | Not Available |
| *A. pipericola* | CHL832 | MT225113 | **MH760542** |
| *A. pipericola* | CHL888 | MT225114 | MT225115 |
| *A. nomius* | NRRL 13137 | **JNOM01000524** | **MH760519** |
| *A. bombycis* | NRRL 26010 | **LYCR01000077** | **LYCR01000200** |
